# Dynamic Changes in Hindlimb Motor Cortex Neurons during Simulated Weightlessness Revealed by Miniature 2-Photon Microscopy

**DOI:** 10.34133/research.0877

**Published:** 2025-09-19

**Authors:** Yuanyuan Fan, Jianwei Li, Jiaying Han, Wenjuan Xing, Yuheng Li, Zizhong Liu, Guohui Zhong, Ruikai Du, Jianguo Zhao, Weijia Sun, Xinxin Yuan, Youyou Li, Hao Yue, Junjie Pan, Xiaoyan Jin, Li Wang, Shukuan Ling, Lifeng Zhang, Yingxian Li

**Affiliations:** ^1^ National Key Laboratory of Space Medicine, China Astronaut Research and Training Center, Beijing, China.; ^2^ School of Life Sciences, Henan University, Kaifeng, China.; ^3^ Department of Aerospace Medicine, Key Laboratory of Aerospace Medicine of the Ministry of Education, Fourth Military Medical University, Xi’an, China.; ^4^ Research Unit of Mitochondria in Brain Diseases, Chinese Academy of Medical Sciences, PKU-Nanjing Institute of Translational Medicine, Nanjing, China.; ^5^ Academy of Advanced Interdisciplinary Study, Peking University, Beijing, China.; ^6^ Department of Physical Education, China Agricultural University, Beijing, China.; ^7^ Research Center for Social Computing and Interactive Robotics, Harbin Institute of Technology, Harbin, China.; ^8^ Oujiang Laboratory (Zhejiang Lab for Regenerative Medicine, Vision and Brain Health), Wenzhou, Zhejiang, China.

## Abstract

Dysfunction of motor behavior during spaceflight is linked to alterations in neuronal activities. However, the longitudinal functional changes in the motor cortex triggered by simulated weightlessness remain ambiguous. In this study, we utilized a miniaturized 2-photon microscope to examine the dynamic shifts in neuronal activities within the hindlimb motor cortex during simulated weightlessness and its subsequent recovery period at the single-cell level. Our results demonstrated that simulated weightlessness led to a progressive decline in motor behavior during open-field and rotarod tasks, which was fully reversed after a 2-week recovery period. Single-cell analysis revealed that hindlimb motor neurons could be classified as activated, inhibited, or unchanged. During active locomotion in the open field, the activity of locomotion-activated neurons increased, while the activity of locomotion-inhibited neurons decreased, despite their numbers remaining constant. Conversely, during passive rotation on the rotarod test, the number of rotation-activated neurons decreased, while their activity increased, and the number of rotation-inhibited neurons increased along with their activity. These changes were largely restored after reloading. These findings elucidate motor dysfunction under simulated weightlessness and the heterogeneous changes in neuronal activities within the hindlimb motor cortex, offering valuable insights into understanding behavioral changes regulated by the motor cortex during spaceflight.

## Introduction

Long-term manned spaceflight has promoted human exploration of space. However, it also presents substantial challenges to the physical and mental health of astronauts. Long-term exposure to the space environment can lead to various physiological changes in astronauts’ bodies, including space motion sickness, muscle atrophy, bone loss, decreased immune function, endocrine dysfunction, and cardiovascular dysfunction [1–3]. Among these, a decline in motor behavior seriously impacts the work efficiency and overall health of astronauts. Studies of real-time human spaceflight have shown a progressive decline in astronauts’ motor behavior during orbital missions, with the most pronounced effect occurring within the first week [4]. Studies of mice transported into space have also shown substantial changes in their motor behavior and gait parameters [5,6]. In addition, studies of the 30-d Bion-M1 satellite mission and simulated weightlessness have shown that mice had reduced distance and range of motor behaviors in open-field tests after spaceflight and decreased latency to fall on the rotarod test [3,7]. However, the precise regulatory mechanisms by which spatial weightlessness affects motor function is currently unclear.

Alterations in motor behavior are intricately linked to changes in the structure and functionality of skeletal muscles and the nervous system [8–12]. Spaceflight can induce muscle atrophy in astronauts, subsequently leading to a decline in motor behavior [13–16]. Recent studies have suggested that during the process of changes in human lower limb strength induced by simulated weightlessness, muscle system atrophy contributes to 39% of the changes, while the central nervous system accounts for 48% [17]. Current research has identified that changes due to the headward distribution of body fluids in a weightless environment include alterations in intracranial free water, increases in ventricular volume, crowding of intracranial apex tissues, and an increase in gray matter density in the motor cortex [18–21]. In terms of brain function, spaceflight can induce extensive changes in cerebral cortical activity [22]. Simulated weightlessness has been found to enhance functional connections between the human right premotor cortex and the bilateral main visual cortex, as well as between the left main motor cortex and the right postcentral gyrus. These enhancements are associated with adaptive changes in motor function [10,11]. Animal experimental studies have demonstrated that simulated weightlessness can induce changes in pathways related to cell stress, inflammation, apoptosis, and metabolism in the brain [23]. There are marked changes in gene expression in multiple brain areas related to motor behavior control, including the motor cortex, striatum, substantia nigra, hypothalamus, and prefrontal cortex [24–26]. The central nervous system plays a crucial role in the changes in motor behavior induced by flight. However, the changing characteristics of neuronal activity in the central nuclei regulating motor behavior have yet to be reported.

The reduction in human motor behavior caused by spaceflight is closely related to changes in lower limb motor function. Research utilizing electrophysiological methods and retrograde tracing has confirmed that the hindlimb motor cortex of the brain regulates both active and passive motor behaviors through a variety of neuronal activity patterns [27–29]. Therefore, it is crucial to monitor neuronal activities in the hindlimb motor cortex in a weightless environment and investigate the regulatory mechanisms influencing changes in motor behavior. Studying the impact of weightlessness on neurons in the motor cortex through observations of active and passive motor behaviors, as well as analyzing changes in neuronal activity under various motor paradigms, is essential for comprehending how the central nervous system functions in a weightless setting. By examining alterations in neuronal activity in a simulated weightless environment and their relationship with motor behavior, we can enhance our understanding of the motor cortex’s regulatory mechanisms in adapting to the challenges presented by spaceflight.

Research examining alterations in hindlimb motor cortex neurons encoding motor behavior during free motion in mice under simulated weightlessness has been constrained by the absence of suitable tools for real-time, single-cell-resolution neuronal activity recording. In this study, we utilized a novel head-mounted micro 2-photon microscope to investigate changes in motor behavior in mice during simulated weightlessness and the subsequent recovery phase of hindlimb unloading (HU). The miniaturized 2-photon microscope (mTPM) offers superior temporal and spatial resolution, deeper penetration (200 to 300 μm), and reduced phototoxicity, enabling us to observe changes in neuronal activity in the motor cortex of freely behaving mice noninvasively and over an extended period [30–32]. This innovative approach enables us to observe heterogeneous changes in neurons within the hindlimb motor cortex under simulated weightlessness conditions. Additionally, we aim to elucidate the patterns and dynamics of neural adaptations in response to both active and passive motor behaviors. These findings will provide critical evidence for understanding the dynamic changes in brain neural functions under spaceflight conditions.

## Results

### Motor behavior dysfunction induced by simulated weightlessness recovered within a 2-week period

**Fig. 1.**
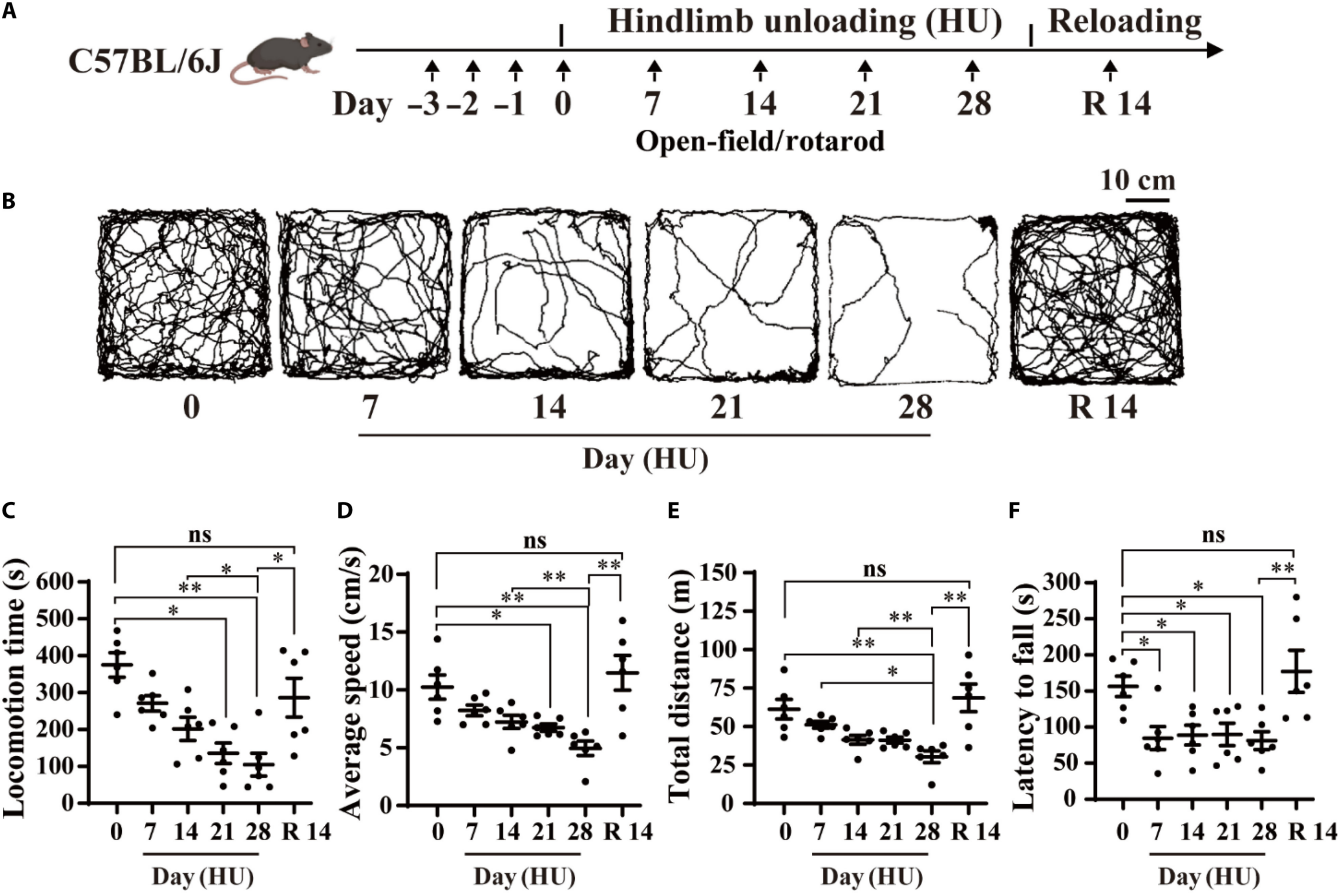
Dysfunctions in motor behavior caused by simulated weightlessness recovered within 2 weeks. (A) Behavioral training and test timeline. Mice were trained to run on a rotarod on days −3 to −1; both rotarod and open-field tests were conducted on days 0, 7, 14, 21, 28, and R 14 (reloading day 14). (B) Representative 10-min trajectories of mice in the open field were recorded on days 0, 7, 14, 21, 28, and R 14 (*n* = 6). (C to E) The locomotion time (C), average speed (D), and total distance (E) of mice during the 10-min recording in the open field on days 0, 7, 14, 21, 28, and R 14 (*n* = 6). (F) The mouse’s latency time to fall from the rotarod on days 0, 7, 14, 21, 28, and R 14 (*n* = 6). Error bars are equal to the standard error of the mean (SEM). The *P* values shown above the graphs were obtained via ordinary one-way analysis of variance (ANOVA) combined with multiple comparison correction. *P < 0.05; **P < 0.01.

While spaceflight may induce disorders in motor behaviors, there is a lack of reported data on the dose–response curve correlating cumulative days spent in space with the effects on motor behavior [9]. The mouse HU model is a widely recognized animal experimental model for simulating weightlessness-induced pathological processes such as microgravity-induced osteoporosis, skeletal muscle atrophy, and neurological dysfunction on the ground [33]. To obtain the temporal changes in motor behavior during simulated weightlessness and its recovery effects, we observed modifications in active locomotion behaviors in the open field and passive rotation behaviors using the rotarod test after 7, 14, 21, and 28 d of simulated weightlessness in mice, as well as the subsequent recovery effect (Fig. 1A). The open-field test is a widely used method to evaluate active locomotion behavior in mice. Figure 1B displays the locomotion trajectories of mice in the open field following simulated weightlessness for varying durations. The results suggest that as the duration of simulated weightlessness increases, the locomotion time, average speed, and total distance gradually decrease in mice (Fig. 1C to E). Following a 14-d recovery period, the locomotion behavior returned to preweightlessness levels (Fig. 1C to E). Additionally, the active locomotion behavior of mice decreases progressively with prolonged simulated weightlessness. Compared to HU for 14 d, a significant reduction in active locomotion behavior is observed in mice after 28 d of simulated weightlessness (Fig. 1C to E).

In the rotarod test, the latency to fall time serves as an indicator of changes in passive acceleration rotation behavior in mice [34]. Following 3 d of rotarod test training, the rotation behavior tends to stabilize in mice. The results indicate that simulating weightlessness for 7 d can cause a decrease in the latency to fall time in mice. After simulating weightlessness for 14, 21, and 28 d, there was no significant difference in the fall latency time compared to the 7-d simulation. Following a 14-d recovery period, the latency to fall time in mice returned to preweightlessness levels (Fig. 1F). The results suggest that simulated weightlessness significantly impacts both active and passive motor behaviors in mice, revealing distinct longitudinal differences. After 28 d of simulated weightlessness, the notable decrease in locomotion behavior compared to the 14-d mark continues to persist. Previous studies have shown that muscle atrophy resulting from HU tends to stabilize after 14 d [35]. Therefore, we hypothesize that the distinct temporal variations in active locomotion and passive rotation behavior response to simulated weightlessness may result from changes in central neuronal activity induced by the simulation of weightlessness.

### Simultaneous miniature 2-photon Ca^2+^ imaging of hindlimb motor cortex L2/3 neurons in freely behaving mice during simulated microgravity

**Fig. 2.**
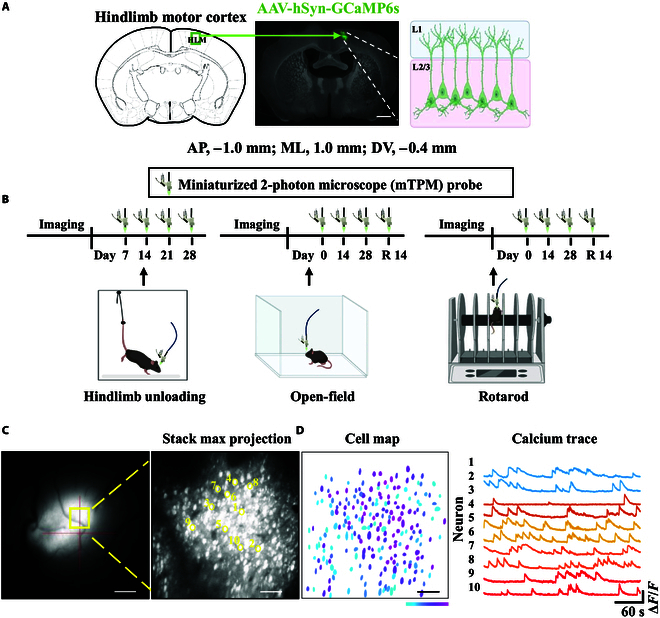
Experimental design using a miniaturized 2-photon microscope (mTPM) to record neuronal activities throughout the pipeline. (A) The inset depicts the mapping of the hindlimb motor cortex (left). Representative image of injection sites marking layer L2/3 in the hindlimb motor cortex (middle). Green, labeled neuronal calcium activity hSyn-GCaMP6s-WPRE-pA virus. Scale bar, 1 mm. Schematic of in vivo 2-photon calcium imaging of layer 2/3 neurons in the hindlimb motor cortex of mice (right). (B) Schematic diagram of the mTPM recording timeline during hindlimb unloading (HU) cage (left), open-field (middle), and rotarod tests in mice (right). (C) Fluorescent GCaMP6s (AAV-hSyn-GCaMP6s) observed under wide-field view. Scale bar, 500 μm (left). A representative stack max projection over 5 min shows GCaMP6s signals in the hindlimb motor cortex. Scale bar, 80 μm (right). (D) The spatial distribution map of active soma identification in neurons, where the color bar represents the intensity of neuronal activity, with purple indicating strong activity and blue indicating weak activity (left). Scale bar, 80 μm. Ten typical representative neuronal calcium traces (right). HLM, hindlimb motor cortex; AP, anteroposterior; ML, mediolateral; DV, dorsoventral.

Although simulated weightlessness may induce motor behavior disorder, the neuronal microcircuit mechanisms underlying motor behavior alteration induced by long-term HU exposure remain elusive. Thus, we combined an mTPM with HU in mice to explore the neuronal activity changes in the hindlimb motor cortex. Mice wearing a head-mounted mTPM were placed in cages and allowed to move freely while monitoring neuronal calcium activity changes in the brain. GCaMP6s (AAV-hSyn-GCaMP6s) was injected into the hindlimb motor cortex region of mice (anteroposterior [AP], −1.0 mm; mediolateral [ML], 1.0 mm; dorsoventral [DV], −0.4 mm) [36,37]. The experiment was performed after the mice fully recovered from surgery within 2 to 3 weeks. Figure 2A shows representative images of GCaMP6s expression in the hindlimb motor cortex. In the following study, we explored the neuronal activity changes in the hindlimb motor cortex of mice after simulated HU for 7, 14, 21, and 28 d. We also evaluated the active and passive motor behavior changes in mice after simulated HU for 0, 14, and 28 d and during a 14-d recovery period. We further explored whether these changes led to cortical circuit dysfunction. The experimental design is shown in Fig. 2B. Finally, we monitored the activity changes of a large population of neurons at single-cell resolution by tracking neuronal activity through GCaMP6s fluorescence expression. Then, the MATLAB software was used to identify target neurons and extract standardized calcium signals for analysis (Fig. 2C).

### Heterogeneous changes in neuronal activities in the hindlimb motor cortex during simulated weightlessness

**Fig. 3.**
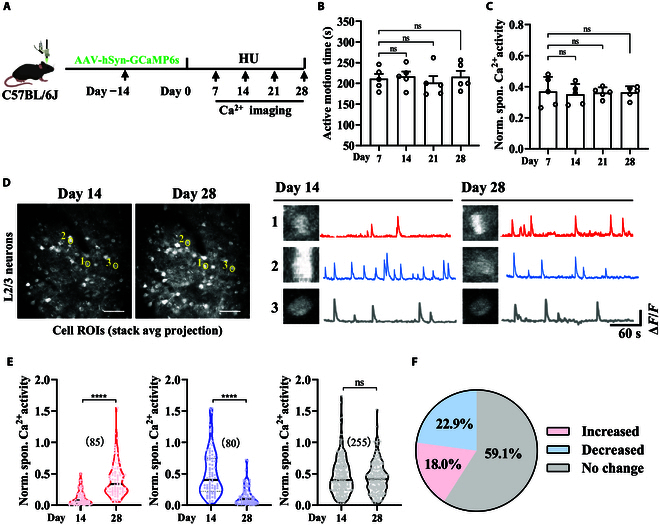
mTPM imaging reveals heterogeneous changes in neuronal activities in the hindlimb motor cortex of mice induced by simulated weightlessness. (A) The experimental timeline to record motor behavior and neuronal activities during simulated weightlessness. (B) During 5 min of HU, the motor behavior of mice remained unchanged (*n* = 5). The active motion time refers to the period during which the mice demonstrate changes in body position within the HU cages. (C) Two-photon imaging was performed for 5 min in the cage. During 5 min of HU, the total neuronal activity in the hindlimb motor cortex of mice remained unchanged (*n* = 5). The normalized spontaneous Ca^2+^ activity was calculated as the time-averaged z-scored Δ*F*/*F*_0_, where Δ*F*/*F*_0_ represents the change in fluorescence relative to the baseline fluorescence (*F*_0_). Error bars are equal to the SEM. The *P* values shown above the graphs were obtained via ordinary one-way ANOVA combined with multiple comparison correction. ns, not significant. (D) Representative average projection fluorescent image of hindlimb motor cortex neurons labeled with GCaMP6s (left). Scale bar, 80 μm. Representative calcium traces from 3 active neurons at different testing stages (right). (E) The pie chart shows the percentage of neurons displaying increased, decreased, or unchanged activity during the long-term HU stage. The number of neurons in each sample size is indicated in the parentheses. Averages are represented by horizontal black lines. Error bars are equal to SEM. *P* values from paired Student *t* tests are indicated above the graphs. ****P < 0.001; ns, not significant. (F) The pie chart illustrates 3 types of neurons with different changes in spontaneous calcium activity after simulated weightlessness (neuron number, 420). Norm. spon., normalized spontaneous; ROIs, regions of interest.

During the 7th, 14th, 21st, and 28th days of simulated weightlessness, we recorded and analyzed the changes in mouse motor behavior and neuronal activity in the hindlimb motor cortex. The experimental procedure is illustrated in Fig. 3A. We placed the mice in a HU cage, acquired GCaMP6s fluorescence from the hindlimb motor cortex using an mTPM, and simultaneously recorded the mice’s motor behavior using a video recording system (Movie S1). The research results suggest that, in line with the patterns of behavioral activity observed in previous studies conducted on mice in the International Space Station [5], the mice’s activity time stabilized after 7 d of simulated weightlessness. Moreover, there were no significant changes in the activity time of the mice in the cages after 7, 14, 21, and 28 d of simulated weightlessness (Fig. 3B). Meanwhile, there were no significant changes observed in the overall calcium activity of neurons within the hindlimb motor cortex as the duration of simulated weightlessness increased (Fig. 3C).

We further tracked the activity changes of 420 neurons in that region at the single-cell level during the simulated weightlessness period ranging from 2 to 4 weeks. The results indicate that as the duration of simulated weightlessness increased, 3 types of neuronal populations in that region exhibited heterogeneous changes. Neurons demonstrating a more than 2-fold increase in the average fluorescence intensity of calcium signals following prolonged simulated microgravity exposure are categorized as “calcium-signal-increased neurons”. Conversely, neurons showing a more than 2-fold decrease in the change in the average fluorescence intensity of calcium signals are labeled as “calcium-signal-decreased neurons”. Neurons failing to meet either of these criteria are classified as “no-response neurons”. Figure 3D shows the locations of 3 typical heterogeneous changes in neurons and their corresponding calcium activity variations from 2 to 4 weeks.

Despite the prolonged duration of simulated weightlessness, there was no significant change in the overall calcium activity of neurons in the hindlimb motor cortex. However, upon conducting single-cell-level analysis of 420 neurons, it was found that 22.9% of neurons exhibited a significant decrease in activity, while 18% showed a significant increase in activity (Fig. 3E and F). By monitoring the activities of individual neurons under long-term simulated weightlessness conditions, it can be observed that with the prolonged duration of simulated weightlessness, the changes in neuronal activities within the hindlimb motor cortex exhibit a sustained disturbance.

### Increased activities in active locomotion-activated neurons during simulated weightlessness and their recovery after reloading

**Fig. 4.**
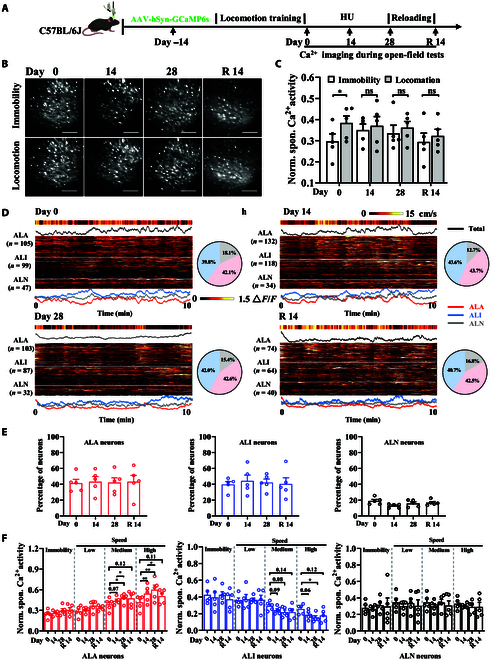
Simulated weightlessness-induced neuronal activity alterations during open field. (A) The experimental timeline to record the changes in neuronal activity induced by long-term HU in mice moving in the open field. (B) The representative average projection of neuronal calcium imaging during immobility (velocity = 0 cm/s) and locomotion (velocity > 0 cm/s) in the open field. Scale bar, 80 μm. (C) Changes in spontaneous calcium activity in mice during immobility (velocity = 0 cm/s) and locomotion (velocity > 0 cm/s) in the open field at different stages of HU (*n* = 5). *P* values from paired Student *t* tests are indicated above the graphs. *P < 0.05; **P < 0.01; ns, not significant. (D) Representative images of the heat maps of calcium activity in mouse ALA (layer 1), ALI (layer 2), and ALN (layer 3) neurons before and after long-term HU (left). The pie chart shows the percentage of 3 heterogeneous changes in neurons (right). ALA, active locomotion-activated; ALI, active locomotion-inhibited; ALN, active locomotion-no-response. (E) Changes in the proportion of the 3 types of neurons before and after long-term HU and during the recovery period in the open-field test (*n* = 5). (F) The changes in the activity of the 3 types of neurons during mouse immobility and locomotion before and after long-term HU and during the recovery period (*n* = 5). Based on real-time video-tracking analysis of spontaneous locomotor activity in the open-field arena, the locomotion velocity of mice was classified into 3 distinct levels, low speed (0 to 5 cm/s), medium speed (5 to 10 cm/s), and high speed (>10 cm/s). Error bars are equal to SEM. The *P* values shown above the graphs were obtained via ordinary one-way ANOVA combined with multiple comparison correction. *P < 0.05; **P < 0.01; ns, not significant.

We placed mice in an open field, recorded their locomotion behavior using a video recording system, and simultaneously monitored the dynamic changes in GCaMP6s fluorescence within the hindlimb motor cortex using an mTPM before simulated weightlessness, on the 14th and 28th days of simulated weightlessness, and during the subsequent 14-d recovery period (Fig. 4A and Video S2). The results indicated that hindlimb motor cortex neurons were more active during locomotion compared to those during immobility before simulated weightlessness. As the duration of simulated weightlessness extended, there was a notable alteration in the speed distribution of mice, characterized by a significant decrease in average speed (Fig. S1) and a disturbance of the neuronal activity pattern, with no activity difference between locomotion and immobility (Fig. 4B and C). Moreover, 14 d of recovery restored locomotion performance to pre-HU baseline levels; the active locomotion-activated (ALA) neuron activities under locomotion and immobility status returned to baseline (Fig. S3A). The results suggest that the spaceflight environment exerts far-reaching effects on brain function.

Further analysis revealed that the neurons exhibited 3 distinct patterns of heterogeneous changes during active locomotion. The first type of neuron showed a significant increase in activity as the locomotion speed increased and is identified as an ALA neuron. The second type of neuron showed no significant change in activity as the locomotion speed increased and is referred to as an active locomotion-no-response (ALN) neuron. Lastly, the third type of neuron exhibited a significant decrease in activity as the locomotion speed increased and is labeled as an active locomotion-inhibited (ALI) neuron. Subsequently, we further analyzed the proportion and calcium activity changes of these 3 types of neurons during different simulated weightlessness periods. Compared to pre-simulated weightlessness, the proportions of these 3 types of neurons did not show significant changes with the extension of the simulated weightlessness duration (Fig. 4D and E). However, after 14 d of simulated weightlessness, there was a significant increase in calcium activity in ALA neurons, while there was a notable decrease in calcium activity in ALI neurons when maintaining the same moderate speed (approximately 5 cm/s) of locomotion. When the mouse’s locomotion speed further increased, reaching high speeds (around 10 cm/s), this increase in fluorescence intensity became even more pronounced (Fig. 4F). Additionally, with further extension of simulated weightlessness, compared to 14 d of simulated weightlessness, after 28 d of simulated weightlessness, there was a further significant increase in calcium activity in neurons when the mouse maintained the same speed in the open field, and after reloading for 14 d, neuronal activity recovered (Fig. 4F). These results suggest that exposure to simulated weightlessness alters active locomotion behavior in mice, characterized by a significant decrease in both the duration and speed of locomotion in the open field. With prolonged simulated weightlessness duration, more recruitment of neuronal activity within the hindlimb motor cortex is required to sustain mouse locomotion behavior in the open field, most obviously at higher speeds.

### Both the number and activity of passive locomotion-related neurons were changed during simulated weightlessness and their delayed recovery

To investigate the neuronal activity changes associated with alterations in the passive rotation behavior of mice under simulated weightlessness of different durations, we positioned mice with an mTPM head-mounted on a rotarod. We then observed their passive rotation behavior and examined the corresponding changes in calcium activity within the hindlimb motor cortex. Initially, we conducted a 3-d training session for this group of mice on the rotarod to minimize the influence of passive rotation behavior variability and neuronal activity on the simulated weightlessness effect [38]. In terms of behavior, the duration spent on the rotarod during the training phase of the rotarod test demonstrated that the mice exhibited rapid motor skill learning ability on the 1st day. As training progressed, their time on the rotarod increased significantly. By the 2nd day, their motor skills had reached a peak level and gradually stabilized. On the 3rd day, the performance remained relatively stable, indicating that after 3 d of training, the mice had effectively mastered the motor task (Fig. S3B). During the acceleration phase of the rotarod task, neuronal activity in the hindlimb motor cortex exhibited a prominent increase in average fluorescence intensity. The effect was particularly evident on the 1st day of motor learning, when most of neurons in this region showed a rapid and robust activation in response to rotarod acceleration, and subsequently decreased sharply upon cessation of the acceleration (Fig. S3A). However, no substantial differences in average fluorescence intensity were observed between the 2nd and 3rd days, suggesting that as the mice became proficient in the task, neuronal activity also reached a stable state (Fig. S3C and D). These findings suggested that during the early stages of motor skill learning, the overall neuronal activity increased substantially due to the involvement of a large population of neurons, including neurons not directly associated with the task. Once the motor skill was acquired, activity was retained primarily in neurons specifically involved in motor control. As a result, neuronal activity becomes selectively expressed and recorded in circuits directly related to motor behavior.

**Fig. 5.**
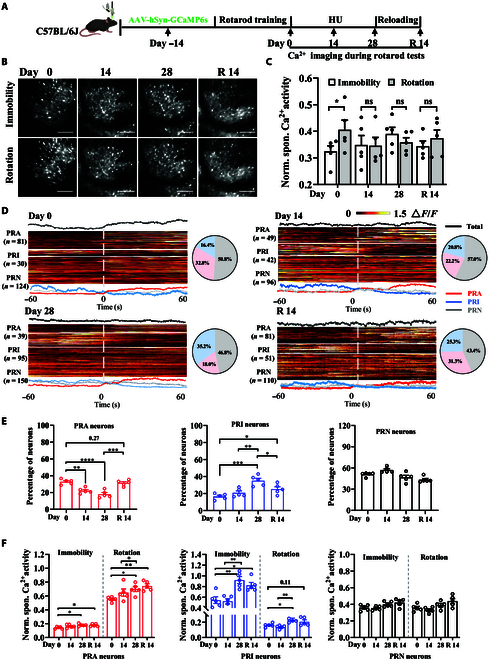
Simulated weightlessness-induced neuronal activity alterations on rotarod. (A) The experimental timeline to record the changes in neuronal activity induced by long-term HU in mice running on the rotarod. (B) Representative average projection of neuronal calcium images during immobility and rotation on the rotarod. Scale bar, 80 μm. (C) Changes in spontaneous calcium activity in mice during immobility and rotation on the rotarod on different stages of HU (*n* = 5). *P* values from paired Student *t* tests are indicated above the graphs. *P < 0.05; ns, not significant. (D) Representative images of the heat maps of calcium activity in mouse PRA (layer 1), PRI (layer 2), and PRN (layer 3) neurons before and after long-term HU (left). The pie chart shows the percentage of 3 heterogeneous changes of neurons (right). PRA, passive rotation-activated; PRI, passive rotation-inhibition; PRN, passive rotation-no-response. (E) Changes in the proportion of the 3 types of neurons before and after long-term HU and during the recovery period the in rotarod test (*n* = 5). (F) The changes in the activity of the 3 types of neurons during mouse immobility and rotation before and after long-term HU and during the recovery period (*n* = 5). Error bars are equal to SEM. The *P* values shown above the graphs were obtained via ordinary one-way ANOVA combined with multiple comparison correction. *P < 0.05; **P < 0.01; ***P < 0.005; ****P < 0.001; ns, not significant.

Subsequently, we monitored the alterations in passive rotation behavior and neuronal activity prior to simulated weightlessness, after 14 d of simulated weightlessness, after 28 d of simulated weightlessness, and during the 14-d recovery period. The experimental procedure is visually depicted in Fig. 5A. We placed the mice on a rotarod, acquired GCaMP6s fluorescence from the hindlimb motor cortex using an mTPM, and simultaneously recorded the mice’s rotation behavior using a video recording system (Video S3). The findings revealed that control mice exhibited a significant increase in neuronal activity during the passive rotation test on the rotarod. However, following 14 and 28 d of simulated weightlessness, no significant alterations in neuronal activity were observed during the passive rotation test on the rotarod. After a 14-d recovery period from simulated weightlessness, rotarod tests revealed that neuronal activity associated with passive rotation behavior in mice had not returned to baseline levels (Fig. S3B). These results further confirm that the spaceflight environment can induce long-term persistent changes in brain neural function.

Upon further analysis, it was discovered that passive rotation behavior led to 3 distinct heterogeneous changes in cortical neurons. The activity of the first type of neuron decreased significantly during passive rotation behavior, leading us to classify them as passive rotation-inhibition (PRI) neurons. The second type of neuron showed no significant changes in activity during passive rotation behavior, hence designated as passive rotation-no-response (PRN) neurons. Lastly, the third type of neuron exhibited a significant increase in activity during passive rotation behavior, and we denoted them as passive rotation-activated (PRA) neurons. Subsequently, we conducted a further analysis of the calcium activity and ratio changes in these 3 types of neurons across varying durations of simulated weightlessness. During the 14-d simulated weightlessness period, the proportion of PRI neurons in the hindlimb motor cortex of mice significantly increased, while the proportion of PRA neurons significantly decreased, with no significant change observed in the PRN neuron proportion. As the simulated weightlessness period was further extended to 28 d, the proportion of PRI neurons further increased, and the proportion of PRA neurons further decreased compared to those for the 14-d period. After 14 d of reloading, the proportion of neurons recovered (Fig. 5D and E). Additionally, after 14 d of simulated weightlessness, there was an increasing trend in the activity of all 3 types of neurons. However, after 28 d of simulated weightlessness, the calcium activity of PRA and PRI neurons significantly increased in mice on the rotarod, whether in static or accelerated states. After 14 d of reloading, neuronal activity recovered (Fig. 5F). These results suggest that exposure to simulated weightlessness leads to changes in passive rotation behavior in mice, manifested by a significant reduction in the time spent on the rotarod. With the prolonged duration of simulated weightlessness, more neuronal activity recruitment within the hindlimb motor cortex was observed during the accelerated running on the rotarod.

### HU affects neuroendocrine regulation and neuroinflammation-related gene expression in the motor cortex

**Fig. 6.**
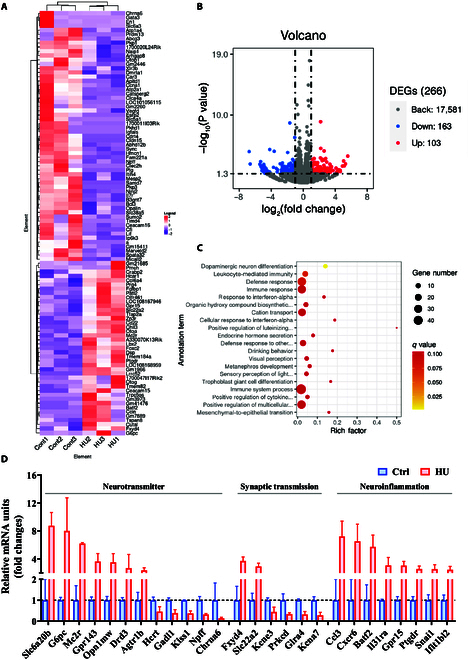
Transcriptomic characteristic changes in the motor cortex under simulated weightlessness. (A) Cluster heat map of differentially expressed genes. (B) Volcano plot of differentially expressed genes (red dots represent up-regulated genes, blue dots represent down-regulated genes, and gray dots represent genes with no significant difference). (C) Bubble plot of Gene Ontology (GO) functional enrichment analysis (the size of bubbles indicates the number of genes, and the color represents the −log_10_(*q* value)). (D) Changes in gene expression related to neurotransmitters, synaptic transmission, inflammation, and immunity. DEGs, differentially expressed genes; mRNA, messenger RNA.

However, it remains unclear whether there are specific alterations at the molecular level and in neural network functional connectivity. Thus, we observed the transcriptome changes in the motor cortex of mice after 28 d of simulated weightlessness. The results showed that compared with the control group, a total of 266 differentially expressed genes were detected in the motor cortex of mice in the HU group (Fig. 6A), among which 103 genes were up-regulated and 163 genes were down-regulated (Fig. 6B); functional enrichment analysis indicated that these differentially expressed genes were mainly enriched in pathways related to dopamine neuron differentiation, leukocyte-mediated immune response, etc. (Fig. 6C). The results showed that in the hindlimb motor cortex of the simulated weightlessness group, the changes were related to biological processes such as neural signal transmission and neuroendocrine regulation. Genes related to the neurotransmitter signaling pathway, such as *Gpr143*, *Mc2r*, and *Agtr1b*, were up-regulated, while *Hcrt*, *Gadl1*, and *Npff* were down-regulated (Fig. 6D). Besides neurotransmitter and hormone regulation, genes related to synaptic transmission and plasticity, such as *Drd3*, *Kcna7*, *Kcne3*, and *Prkcd*, were down-regulated following HU-induced simulated weightlessness, indicating alterations in neural signal transmission. Simulated weightlessness also caused a marked activation of neuroinflammation in the motor cortex, evidenced by up-regulation of genes including *Ccl3*, *Cxcr6*, *Il31ra*, and *Gpr15*, suggesting that HU induces neuroinflammation in the motor cortex.

## Discussion

**Fig. 7.**
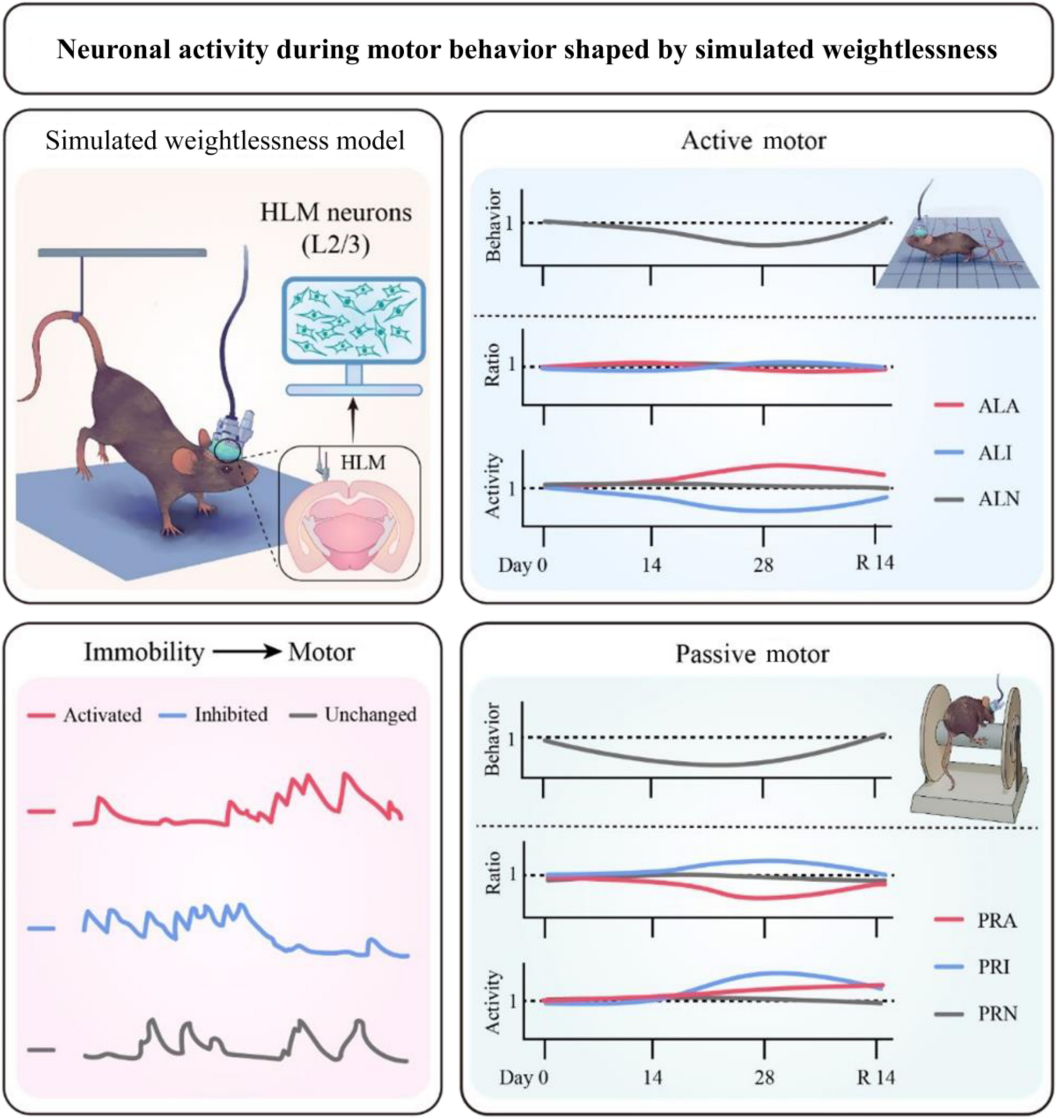
The dysfunctions of motor behavior and brain functions caused by simulated weightlessness recovered asynchronously. This figure illustrates changes in neuronal activity in the hindlimb motor cortex during disrupted motor behavior in a simulated microgravity environment (top left). In the hindlimb motor cortex, 3 different types of neuron populations—active neurons, inhibitory neurons, and unchanged neurons—exhibit distinct responses to motor behaviors (bottom left). Prolonged HU disrupts motor behaviors in mice and the dynamic activity of these 3 neuron populations. As the duration of simulated weightlessness increases, motor behavior continues to decline (top left). Long-term simulated microgravity induces heterogeneous alterations in neuronal activity. Furthermore, these changes can be partially reversed after a 14-d recovery period, improving cortical neuron dysfunction (top right). However, there is a mismatch between the extent of neuronal function recovery and the recovery of motor behavior. L2/3 refers to layers 2 and 3 of neurons in the hindlimb motor cortex. ALA represents active locomotion-activated neurons, ALI refers to active locomotion-inhibition neurons, and ALN denotes active locomotion-no-response neurons. Similarly, PRA stands for passive rotation-activated neurons, PRI for passive rotation-inhibition neurons, and PRN for passive rotation-no-response neurons.

In this study, we systematically elucidated the longitudinal changes in motor behaviors and neuronal calcium activities in the hindlimb motor cortex of C57BL/6J mice under simulated weightlessness and following recovery. We found that as the duration of simulated weightlessness increased, there were continuous disorders in motor behaviors and neuronal activities (Fig. 7). In detail, with the prolonged duration of simulated weightlessness, motor function continuously declined, and single-cell analysis revealed heterogeneous changes in the proportion and magnitude of neuronal activities over time. Two weeks’ recovery restored all behavioral performances, with brain functional alterations partially rehabilitated. Motor behaviors and brain functions presented a mismatch during recovery period.

Simulated weightlessness can lead to motor dysfunction and postural control impairment. Using a 10° head-down tilt bed rest model to simulate weightlessness, Clément’s team [39] found that just 5 d of exposure impaired postural control sufficiently that a mere 25 min of standing induced balance dysfunction. These neuromotor disturbances stem from disrupted coordination between central nervous system processing and peripheral musculoskeletal function. Importantly, subsequent analyses identified the hindlimb motor cortex as critically involved in this adaptive response, where specialized neuronal populations exert precise control over hindlimb motor and postural maintenance [40]. Spaceflight-induced muscle atrophy typically peaks around the 14th day [35,41]; however, it remains unknown whether motor behavior continues to decline after 14 d. Our research found that as the simulated weightlessness time prolongs, the active locomotion behavior in the open field continues to decline after 28 d of simulated weightlessness compared to locomotion behavior at 14 d. It is speculated that in addition to the effect of muscle atrophy on the behavior of mice, the impact of simulated weightlessness on the central nervous system, particularly the hindlimb motor cortex, also plays an important role. In the active locomotion experiment, it was found that the activity of activated neurons in the hindlimb motor cortex during high-speed locomotion was significantly higher after 28 d of simulated weightlessness than after 14 d. This indicates that with the prolonged duration of simulated weightlessness, more neuronal activity is required to sustain high-speed motor behavior. As for the passive rotation behavior during the rotarod test, there was no difference in rotation behavior between the 14th and 28th days of HU. However, it was found that the activity and proportion of neurons in the hindlimb motor cortex encoding rotation behavior changed, confirming that neuronal activity in the hindlimb motor cortex underwent persistent changes after simulated weightlessness. These findings reveal the complex dynamic changes in neural activity within the hindlimb motor cortex under simulated weightlessness conditions and their important role in the altered motor function induced by simulated weightlessness. With prolonged simulated weightlessness, the diverse alterations in neuronal activity during motor behavior imply a sustained influence of simulated weightlessness on neural circuits governing motor control. This underscores an adaptive mechanism embraced by the brain to navigate extended periods in simulated weightlessness environments. These phenomena lay the groundwork for comprehending the neurobiological underpinnings of motor regulation during spaceflight and strategies for adapting to the challenges of the space environment.

Previous studies utilized electrophysiological methods to observe the characteristics of hindlimb motor behavior encoded by the hindlimb motor cortex in rodents, cats, and primates [27–29]. Compared to passive running of mice on a treadmill, free running requires more neuronal activity in the hindlimb motor cortex [40], suggesting substantial differences in the activity characteristics of motor cortex neurons during active and passive motor behaviors. In our study, we found that the neural discharge patterns of motor cortex neurons can be categorized into 3 types when involved in regulating active and passive motor behaviors. Furthermore, the neural discharge patterns involved in regulating these 2 types of motor behaviors exhibit different responses to simulated weightlessness. There were notable differences in the neuron populations recruited in the motor cortex during the changes in the 2 types of motor behaviors caused by simulated weightlessness. This indicates a close relationship between changes in these neuron populations and the altered forms of motor behavior observed under simulated weightlessness. We observed that the passive rotation behavior of mice no longer continued to decline as the simulated weightlessness time increased, but the proportion of different reactive neuron activities in the motor cortex before and after acceleration still changed continuously. This showed a sustained increase in the proportion of PRI neuron activity and a sustained decrease in the proportion of PRA neuron activity. Simulated weightlessness has a relatively small effect on the proportion of active locomotion-related response neurons in the motor cortex, but it does affect the activity of ALI and ALA neurons, leading to a sustained decrease in locomotion behavior in the open field. These results indicate that with the increasing duration of simulated weightlessness, heterogeneous changes occur in the activity patterns of neurons within the hindlimb motor cortex, which are involved in regulating various behavioral activities. This adaptation aims to maintain and accommodate the changing demands of various behavioral capabilities during spaceflight.

The brain dysfunctions caused by simulated weightlessness restored gradually during the reloading period, with a slower rehabilitation speed compared to motor behavioral performances. Previous studies have also shown that spaceflight can lead to changes in brain structure, function, neurotrophic factors, pro-inflammatory cytokines, chemokines, oxidative stress, and protein misfolding, as well as differential expression of numerous genes within the brain [23,42,43]. These microenvironmental disturbances may require a longer period to recover. Structurally, prolonged exposure to microgravity can cause changes in certain brain structures such as gray matter, white matter, fiber tracts, and the redistribution of cerebrospinal fluid [9,44–49]. These structural changes necessitate extended time to return to normal conditions. Brain neuronal activity relies on complex network connections and signal transmission [50], which microgravity can disrupt, delaying the recovery process. Additionally, the function of the blood–brain barrier may be compromised under microgravity conditions [51], impairing the ability of neurons to obtain essential nutrients and oxygen, and thus may slow down the recovery of neuronal activity. Spaceflight may also impact the regeneration and repair capabilities of neural cells [52], a process that is typically slower in the complex microgravity environment, which may further delay neuronal activity recovery. Regarding brain function, psychological stress and emotional fluctuations are critical factors that negatively impact neuronal activity. Moreover, the delayed recovery of brain neuronal activity compared to motor behavioral function recovery is the result of multiple interacting factors. These factors include changes in brain structure, the complexity of neuronal connections, alterations in the blood–brain barrier, disruptions in neurotransmitter balance, psychological and emotional factors, lack of gravity stimulation, sleep quality, and the processes of neuronal regeneration and repair. Research needs to further investigate these factors to develop more effective interventions aimed at promoting the comprehensive recovery of brain function.

Our study demonstrated that under simulated weightlessness conditions, the motor cortex undergoes changes in genes related to dopaminergic neurodifferentiation, synaptic transmission and plasticity, and neuroendocrine regulation. Notably, the dopaminergic signaling pathway, a crucial neuromodulatory system for motor control, shows distinct alterations in gene expression. At the molecular level, spaceflight can trigger changes in neurotransmitter levels, including alterations in neurotrophic factors, pro-inflammatory cytokines, chemokines, and oxidative stress. These changes may further affect the recovery of neuronal activity. Moreover, our findings indicate that HU induces substantial enrichment of immune inflammation-related pathways in the motor cortex, significantly increasing the expression of neuroinflammation-related genes such as *Ccl3* and *Il31ra*. It has been established that 30 d of simulated microgravity through HU leads to increased apoptosis, oxidative stress, and neuroinflammation in the brain, along with changes in cerebral gene expression profiles associated with microgravity adaptation and fluid redistribution [53–55]. These results suggest that HU can trigger neuroinflammation in the motor cortex, which contributes to functional changes in neurons in this region.

Changes in motor behavior are not only related to the remodeling of musculoskeletal functions caused by weightlessness but also closely associated with the adaptive changes in the central nervous system. Existing studies have used the HU model to replicate certain behavioral changes associated with spaceflight, such as cognition and motor impairments, confirming that this model can be used in ground-based research to simulate the effects of microgravity on the central nervous system [6,7]. Actual spaceflight’s weightlessness causes fluid redistribution, leading to remodeling of cerebral arteries, such as increased vasodilation and reduced vascular stiffness [8–10]. Similarly, our previous research showed that HU induces carotid artery thickening and fluid redistribution [11], supporting the model’s ability to partially replicate the hemodynamic changes associated with spaceflight. Additionally, since the isolation in the HU model could also affect motor behavior and neural function, in our study, mice underwent a 2-week adaptation period to minimize stress from individual housing before testing. Baseline data for each mouse prior to the HU model were used as a control, ensuring that the observed differences primarily resulted from the simulated weightlessness induced by HU.

In the study, our findings disclosed that the neurons within the hindlimb motor cortex exhibited heterogeneous changes under simulated weightlessness, and certain neuronal activity still had not returned to baseline levels throughout the recovery period. We also observed that locomotion behavior disorders resulting from simulated weightlessness are linked to changes in neuronal activity in the hindlimb motor cortex. The motor cortex contains various types of neurons involved in regulating motor functions, each with distinct roles in the modulation of locomotion. While there is no direct molecular or circuit-level explanation for why specific neuron subtypes respond heterogeneously to HU, further investigation is needed to explore the plastic changes in different neuron types during weightlessness, alterations in functional connectivity within neural circuits, and their effects on locomotion. In summary, our study offers new insights into the plasticity mechanisms of neuronal activity adapting to simulated weightlessness environments and lays a foundation for understanding the regulation of locomotion functions during actual spaceflight.

## Materials and Methods

### Animal model

Simulated weightlessness achieved through HU in mice is a widely utilized method in space medicine research [56]. In this study, adult male C57BL/6J mice aged 8 to 10 weeks underwent HU for durations of 14 and 28 d. Prior to and throughout the entire experiment, adult male C57BL/6J mice were consistently housed individually in cages. Meanwhile, they had free access to food and water. The HU was carried out at a 30° angle, allowing the mouse’s hindlimbs to hang freely in the air while its forelimbs remained on the ground. During the open-field and rotarod tests, the mice were removed from the HU cages to undergo behavioral assessments. In the open-field test, the mice were tested once per week for 10 min each session. In the rotarod test, the testing duration for the mice was less than 5 min. The total daily testing time per day did not exceed 30 min. All animal experiments were conducted in a sterile breeding environment, adhering to approved protocols by the Animal Care Committee of PKU-Nanjing Institute of Translational Medicine (Approval ID, IACUC-2021-023).

### Open-field test

The voluntary exercise behavior of mice was assessed in a 50 × 50 × 30 cm open field. The same experimenter conducted the study. First, mice were acclimated to the open field for 3 d. After the mice adapted to the open-field test environment for 30 min daily, the experimental test and records were carried out. Next, C57BL/6J mice wearing an mTPM were placed in the test environment for 10 min for monitoring. The above training and tracking were all tested under dim light. After the test, the MiceBehavior software combined with a manual calibration video was used to record and analyze the active locomotion behavior of the mice. The behavioral indicators of the mice, such as locomotion time, average speed, and total distance, can be used to evaluate their active motor functions. The inner zone was defined as the 25 × 25 cm central area of the experiment.

### Rotarod test

Following the open-field test, mice were placed on the rotarod. Two-photon calcium imaging and free-rotation tests were used to simultaneously monitor the changes in passive motor behavior and neuronal activity in the brain after HU. The rotarod acceleration range was set at 4 to 30 rpm/min, and the acceleration time was 300 s (ENV-575M, Med Associates). In the dim light, mice underwent 3 d of accelerated skill learning, with 5 daily tests. The experiment was stopped when the mouse fell from the loading rod or after 300 s of testing, and the mouse had a rest period of 300 s in each experiment. Three days after the adaptive training, the behavior and calcium signal monitoring of HU mice can be carried out. After the test, the changes in passive rotation behavior were assessed by analyzing the time the mice were on the rotarod.

### Virus injection

During the virus injection procedure, the head of the C57BL/6J mouse was fixed onto a stereotaxic apparatus and anesthetized by inhalation of isoflurane (gas flow rate 1 to 1.5 l/min, anesthesia level 5). Eye ointment (Simplex, Actavis) was applied to prevent the eyes from drying out. A hole was drilled in the left hemisphere of the mouse brain, targeting the M1 hindlimb motor cortex (AP, −1.0; ML, 1.0; DV, −0.4) [36,37]. After drilling and exposing the brain surface, the hole was immediately covered with drops of saline (NaCl 9 mg/ml, B. Braun Medical). At the same coordinates as the drilled hole, the adeno-associated virus was injected into the hindlimb motor cortex using a pulled glass micropipette (Cat. No. 504949, World Precision Instruments) at a rate of approximately 50 to 70 nl/min. The virus used was hSyn-GCaMP6s-WPRE-pA (titer, 3.41 × 10^12^ vg/ml, undiluted, Addgene), with an injection volume of 0.3 μl per site. After each injection, the glass pipette was left in place for 10 min to allow the virus to diffuse before retraction. Before conducting the mouse experiments, we allowed the mice to recover for at least 2 weeks postsurgery.

### Mouse craniotomy and postoperative recovery

Before installing the cranial surgery headpiece, erythromycin ointment was applied evenly to the mouse’s eyes to prevent dryness. Then, using a medical cotton swab, approximately 0.2 cm^3^ of depilatory cream was evenly applied to the mouse’s head, continuously wiping to remove hair and expose and clean the skull. Next, using the virus injection site as the center, fine forceps were used to place a circular coverslip of an appropriate size on the skull surface, marking the craniotomy area. Using a skull drill, the skull was drilled clockwise until the entire cranial window was completely severed. If bleeding occurred during the craniotomy, the bleeding site was covered with a gelatin sponge soaked in saline, and then the coverslip was secured with biological tissue glue. The installation of the baseplate and fluorescence screening were performed. When installing the baseplate, an eyelash brush was used to apply a small amount of 502 glue around the circumference of the head cap’s center, securing it to the skull with the cranial window positioned in the center of the head cap. Then, an eyelash brush was used to apply dental cement, filling the gap between the metal head cap and the skull, and the dental cement was allowed to dry. Before fluorescence screening, the mouse to be imaged was fixed onto a mouse adapter, the wound was cleaned, and fluorescence screening was performed. The instrument baseplate was adjusted to the appropriate height and fixed with AB glue. Before conducting any experiments, the mice were allowed to recover for at least 2 weeks postsurgery.

### Miniature 2-photon imaging

Following a period of 3 to 4 weeks for viral expression, the mice were imaged. A baseplate was affixed to the mouse’s head, and a miniature 2-photon microscope holder was secured in place using dental acrylic resin. The miniature 2-photon microscope (FHIRM-TPM V2.0, field of view, 420 × 420 μm^2^; resolution, ~1.13 μm; working distance, 1 mm) is removable, with its stand capable of being installed on the base of the mouse’s head [31]. Prior to imaging, the headpiece should be mounted on the bracket and locked in place with M2 screws. When using a 2-photon microscope, it is usually necessary to mount the microscope (weighing 2.13 g) on the head of a mouse to perform high-resolution imaging in freely moving animals. To counterbalance the weight of the head-mounted 2-photon microscope, we used a pulley system to balance and support the weight of the microscope. Imaging data were captured using imaging software (GINKGO-MTPM, Transcend Vivoscope Biotech Co., Ltd, China) to acquire imaging data at a frame rate of 9.62 Hz (512 × 512 pixels), utilizing a femtosecond fiber laser (~35 mW at the objective, TVS-FL-01, Transcend Vivoscope Biotech Co., Ltd, China). GCaMP6s was excited at 920 nm and imaged using a 520/30 filter.

### Image processing and soma identification

Simultaneous recording of mouse behavior and neuronal calcium signals was achieved. Both neuron videos and behavior videos were recorded synchronously at a sampling frame rate of 9.62 Hz, ensuring that the time point of the neuron corresponded with the time point in the mouse behavior video. The MATLAB software (MathWorks Inc.) preprocessing pipeline was employed for image processing in each 2-photon calcium imaging instance. Initially, the NoRMCorre method was utilized to correct for slight *x*–*y* motion within the field of view caused by mouse motor behavior [57]. Subsequently, the neuronal soma of the neuronal calcium signal was extracted using the neuron soma identification method developed by Professor Zhang’s group [58]. Finally, manual verification was conducted through additional self-developed work to ensure accuracy.

### Calcium signal extraction

Upon identifying the region of interest (ROI), calcium signal extraction was conducted from the identified neurons using the widely recognized ring subtraction algorithm [58,59]. The details are as follows: the calcium signal Δ*F*/*F* of each neuron was calculated as Δ*F*/*F* = (*F*_ROI_ − *α* × *F*_con_)/*F*_0_, where *F*_ROI_ = *F*_raw_ − *F*_background_. *F*_ROI_ is the calcium signal fluorescence intensity minus the background signal in the video. *F*_con_ is the calcium signal of *z*-axis contamination. *F*_raw_ represents the average fluorescence signal within the ROI. *F*_background_ is calculated by selecting a background region adjacent to the ROI of the target neuron, which is free from any target signal, and then computing the average fluorescence intensity of this region as the background fluorescence value (*F*_background_). *F*_0_ is the baseline value of *F*_ROI,_ estimated from the mean value of *F*_ROI_.

### Subgrouping of ALA neurons, ALI neurons, and ALN neurons into functional ensembles

To determine the changes in functional neuronal populations involved in the modulation of active locomotion behavior during simulated weightlessness, we categorized them into 3 groups based on the correlation between neuronal activity in the hindlimb motor cortex and the velocity of active locomotion. We designated these groups as follows: ALA (active locomotion-activated neurons), ALI (active locomotion-inhibitory neurons), and ALN (active locomotion-no-response neurons). The classification algorithm comprised 3 steps: (a) The similarity between the calcium trace (Δ*F*/*F*) *Cn* of each neuron *n* and the behavioral vector *B* of the mouse’s active locomotion was calculated. The similarity was computed using the dot product method, specifically 2*B* · *Cn*/(|*B*|^2^ + |*Cn*|^2^) [58,60]. Similarity values were constrained between 0 and 1, where 1 indicated identical trends between behavior and calcium signals, while 0 indicated complete dissimilarity. (b) Subsequently, for each neuron, the behavioral vector *B* was randomly shuffled, and the similarity between the neuron’s behavioral vector and calcium trace *Cn* was computed. This process was repeated 5,000 times to generate a histogram representing the distribution of similarity predicted by random shuffles for the given neuron. (c) Neurons were classified as ALA neurons if their actual similarity exceeded 99.17% of the similarity distribution histogram generated from random shuffling. Conversely, neurons were defined as ALI neurons only if their actual similarity fell below 0.83% of the similarity distribution histogram generated from random shuffling. Neurons that did not meet either of these conditions were classified as ALN neurons.

### Subgrouping of PRA neurons, PRI neurons, and PRN neurons into functional ensembles

To identify functional neuronal populations critical to HU-induced rotation behavioral disorders, we categorized neurons in this region into 3 classes of neurons with distinct reactivity. The ratio changes of the calcium signal of each neuron before and after the acceleration of the mouse was calculated as ratio = speed (avg Δ*F*/*F*)/stop (avg Δ*F*/*F*). A PRA neuron is defined as a neuron whose ratio is more than 2 times the baseline average fluorescence intensity after the accelerated rotation of the mouse. A PRI neuron is defined as a neuron whose fluorescence intensity drops below 0.5 times the baseline average after the mouse undergoes accelerated exercise. Neurons that do not meet either of the above criteria are defined as PRN neurons. A PRN neuron is defined as the absence of alterations in the average fluorescence intensity of calcium signals within neurons following accelerated mouse rotation.

### Calculation of the event average Δ*F*/*F* calcium traces

We examined the longitudinal alterations in the calcium signals of neuron groups exhibiting varied responses during motor behavior disorders induced by simulated weightlessness at a single-cell level. We determined the characteristics of the average Δ*F*/*F* changes in neuron groups with distinct responses and the total neuron population in mice across 2 motor behavior events. Specifically, in the open-field experiment, we assessed the average Δ*F*/*F* changes of ALA, ALI, and ALN neurons and the total neuron population in the hindlimb motor cortex of mice at various stages of simulated weightlessness during a 10-min active motor behavior period in the open field. In the rotarod test, we evaluated the average Δ*F*/*F* changes of PRA, PRI, and PRN neurons, as well as the total neuron population in the hindlimb motor cortex of mice at different stages of simulated weightlessness before and after accelerated rotation on the rotarod. The calcium signal Δ*F*/*F* of each neuron was smoothed using a low-pass filter with a span of 5 to derive the trigger signal of the neuron event, and the average Δ*F*/*F* of the neuron calcium signal in different activity states of the mouse was calculated.

### Histological analysis

After deep anesthesia with pentobarbital sodium, the mice were perfused and fixed with 4% paraformaldehyde via the cardiac vein. The brain was removed and fixed in 4% paraformaldehyde overnight at 4 °C and then transferred to 30% sucrose solution at 4 °C for 2 d; 40-μm serial frozen coronal sections were prepared. Brain slices were washed 3 times in PBST (phosphate-buffered saline, 1% Triton X-100) for 10 min. After mounting the slides, the injection site was confirmed by an FV-1200 confocal laser scanning microscope, and images were analyzed using ImageJ (National Institutes of Health, USA).

### Statistical analysis

All data are presented as mean ± standard error of the mean. Graphing and statistical analysis were performed using Prism 8.1 (GraphPad) or MATLAB. Two-group comparisons were calculated using the Student *t* test (2-tailed, paired). One-way repeat measures analysis of variance (ANOVA) was used to analyze the data for multiple group comparisons. When data meet the assumptions of normality and homogeneity of variance, one-way ANOVA with Tukey’s post hoc test is appropriate for comparing group means. If these assumptions are violated, the Kruskal–Wallis test is used, followed by Dunn’s test for multiple comparisons. A *P* value <0.05 indicates statistical significance. Note that the Kruskal–Wallis test assumes similar distribution shapes across groups, and Welch’s ANOVA is preferred when data are normal but heteroscedastic.

## Data Availability

The data supporting the findings of this study are available from the corresponding authors upon reasonable request.
