# Supplementary material for: Dynamic Changes in Hindlimb Motor Cortex Neurons during Simulated Weightlessness Revealed by Miniature 2-Photon Microscopy
Source: Research (Wash D C). 2025 Sep 19;8:0877. doi: 10.34133/research.0877 (PMC12446757; doi:10.34133/research.0877)
Supplement: Supplementary 1 — Figs. S1 to S3 Movies S1 to S3 [file research.0877.f1.zip › SUPPLEMENTARY MATERIALS.docx]

SUPPLEMENTARY MATERIALS


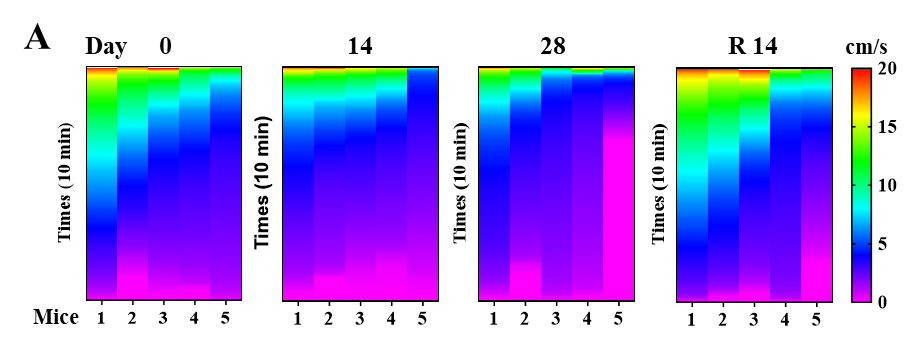


Figure S1. In the open field test, the decrease in locomotion speed caused by simulated weightlessness in mice recovered within two weeks. A. Mouse locomotion speed distribution heatmap during 10-minute open field test (n=5).


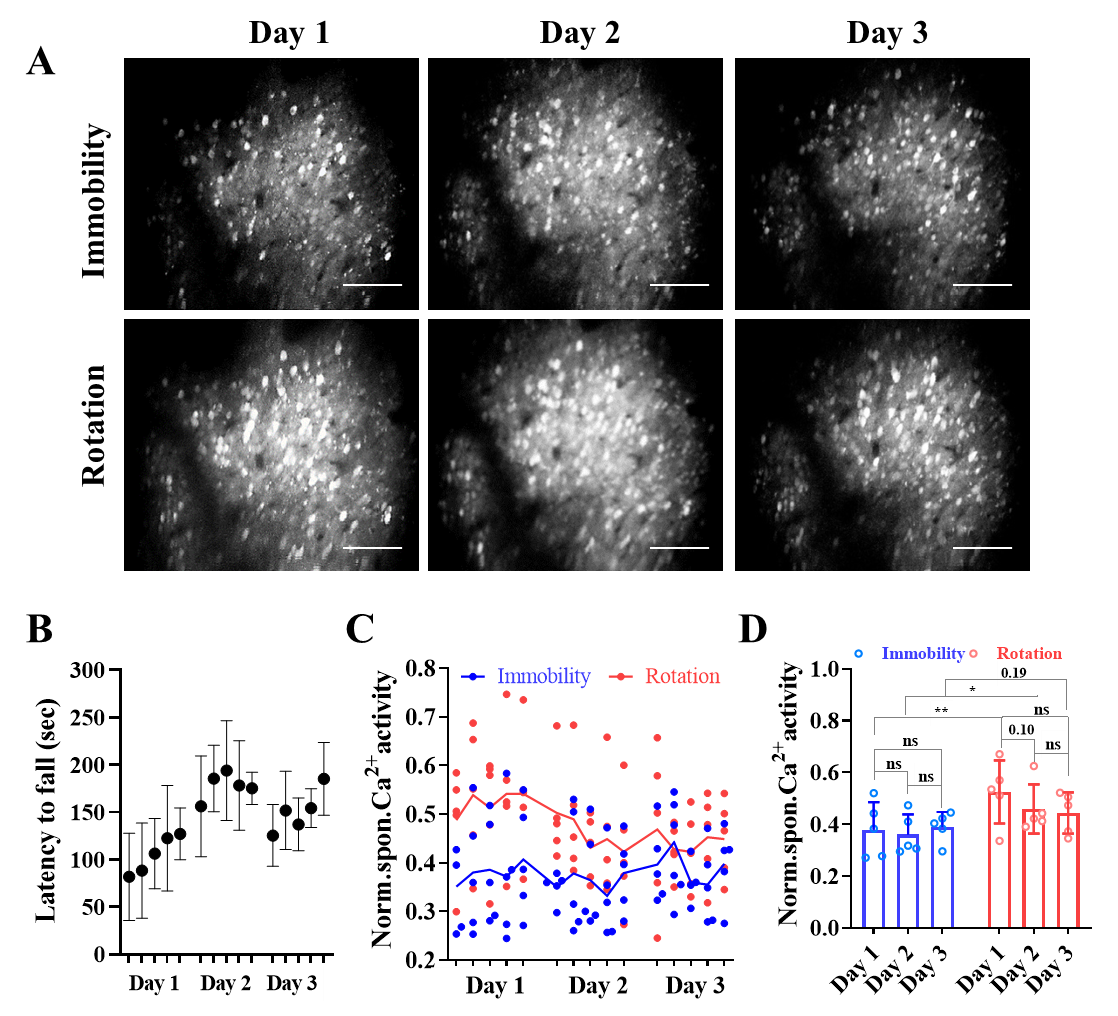


Figure S2. Behavioral and calcium signal changes in mice during rotarod training. A. Representative images showing the average calcium activity of neurons in mice during immobility (up) and rotation (down) states over three days of rotarod adaptation training. Scale bar: 100 μm. B. Changes in the time mice stayed on the rotarod over three days of rotarod adaptation training. ​C. Trend graph showing the changing trends of average calcium activity in neurons during immobility (blue dots) and rotation (red dots) states in mice over three days of rotarod adaptation training​. D. Statistical graph showing the changes in average calcium activity of neurons during immobility (blue dots) and rotation (red dots) states in mice over three days of rotarod adaptation training. Error bars are equal to SEM. The *P*-values shown above the graphs were obtained via ordinary one-way ANOVA combined with multiple comparison correction. **P* < 0.05. ***P* < 0.01. ns, not significant.


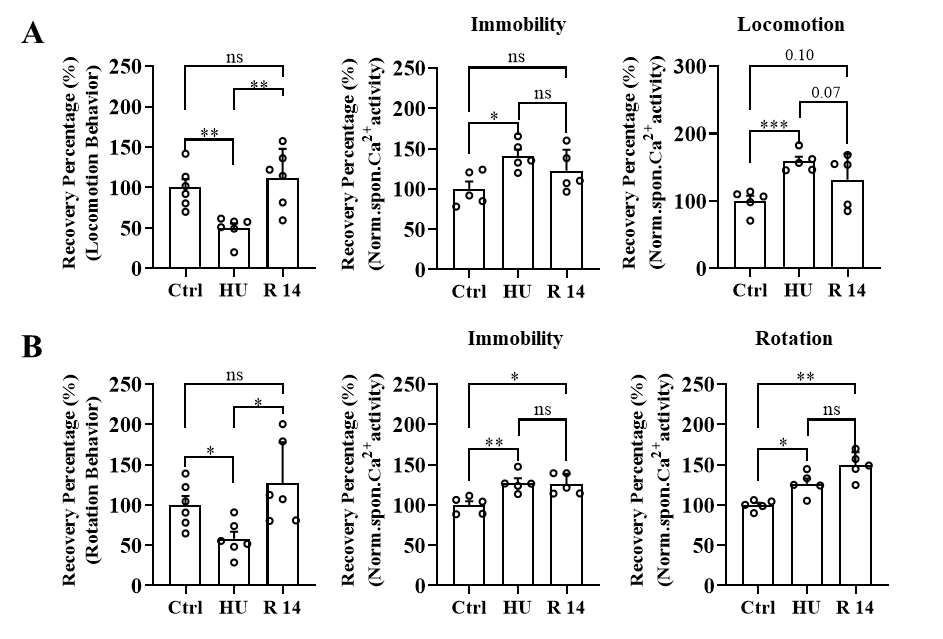


Figure S3. Percentage of recovery of motor behavior and neural activity to baseline level. A. Open field test showing the percentage recovery of locomotion behavior and neuronal activity to baseline levels in mice. Left, Percentage recovery of locomotion behavior; Middle, Percentage recovery of ALA neuronal activity during locomotion; Right, Percentage recovery of ALA neuronal activity during locomotion. B. Rotarod test showing the percentage recovery of rotation behavior and neuronal activity to baseline levels in mice. Left, Percentage recovery of rotation behavior; Middle, Percentage recovery of PRA neuronal activity during immobility; Right, Percentage recovery of PRA neuronal activity during rotation. Error bars are equal to SEM. The *P* values shown above the graphs were obtained via ordinary one-way ANOVA combined with multiple comparison correction. **P* < 0.05, ***P* < 0.01, ****P* < 0.001, ns, not significant.

Movie S1. Simultaneous display of mouse motor behavior, GCaMP6s recording grayscale video, and identified neuronal calcium transients for a representative C57BL/6J mouse in a hindlimb unloading cage. Upper Left Panel: A freely moving mouse with a head-mounted two-photon miniature microscope in a hindlimb unloading cage. Upper Right Panel: Motion-corrected GCaMP6s calcium grayscale video captured by a two-photon miniature microscope during mouse motor behaviors. Lower Panel: Calcium activity trace of the relevant neuron identified within the field of view during a 5-minute time window.

Movie S2 Simultaneous display of mouse locomotion, GCaMP6s recording grayscale video, and identified neuronal calcium transients for a representative C57BL/6J mouse in the open field. Upper Left Panel: A freely moving mouse equipped with a head-mounted two-photon miniature microscope in an open field. Upper Right Panel: Motion-corrected GCaMP6s calcium grayscale video captured by a two-photon miniature microscope during free locomotion in the open field. Middle Panel: Representative images displaying heat maps of mouse speed. Lower Panel: Calcium activity trace of the relevant neuron identified within the field of view during a 10-minute time window.

Movie S3 Simultaneous display of mouse rotation, GCaMP6s recording grayscale video, and identified neuronal calcium transients for a representative C57BL/6J mouse on the rotarod. Upper Left Panel: A freely rotating mouse equipped with a head-mounted two-photon miniature microscope on a rotarod. Upper Right Panel: Motion-corrected GCaMP6s calcium grayscale video captured by a two-photon miniature microscope during mouse rotation. Lower Panel: Calcium activity trace of the relevant neuron identified within the field of view during a latency to fall time window.
